# *Fusobacterium nucleatum*: a rare cause of pyogenic liver abscess

**DOI:** 10.1186/s40064-015-1090-8

**Published:** 2015-06-19

**Authors:** Sajan Jiv Singh Nagpal, Dhruvika Mukhija, Preethi Patel

**Affiliations:** Department of Internal Medicine, Cleveland Clinic, Cleveland, OH 44195 USA; Department of Hospital Medicine, Cleveland Clinic, Cleveland, OH 44195 USA

**Keywords:** Pyogenic liver abscess, Fusobacterium, Poor dental hygiene, Immunocompromised state

## Abstract

While pyogenic liver abscesses are uncommon, they are associated with significant mortality and morbidity. Most pyogenic liver abscesses are polymicrobial and are caused by enteric bacteria and anaerobes. Rarely, mono-microbial infections may occur, typically in immunocompromised individuals. We report the unusual case of a 69 year-old immunocompetent female who developed a pyogenic liver abscess due to *Fusobacterium nucleatum* infection, likely from a dental source. Poor oropharyngeal hygiene seems to have a major role in infection from this organism and therefore *F. nucleatum* should be considered as a differential for causes of pyogenic liver abscess in such patients. Drainage of the abscess and antibiotic therapy are the mainstays of therapy.

## Introduction

Worldwide, amebic liver abscess remains the most common form of liver abscess, but in the United States pyogenic liver abscess (PLA) is the most common form (Wong et al. 2002). The incidence of pyogenic liver abscesses is estimated at about 2.3 per 100,000 patients (Huang et al. 1996; Kaplan et al. 2004) and mortality is estimated at 2–12% (Mohsen et al. 2002; Rahimian et al. 2004) despite advances in antibiotic therapy and investigation and management of these abscesses over the years.

Due to its rich blood supply from the portal and systemic circulations, the liver is a common site of metastatic disease and also the most common site of visceral abscesses. According to a large case series, an estimated 48% of intra-abdominal abscesses involve the liver (Altemeier et al. 1973). Intra-abdominal infections, particularly those involving the biliary tract are more likely to lead to the formation of a PLA (Huang et al. 1996; Rahimian et al. 2004). Bacteria may also spread through the portal vein or from hematogenous seeding from bacteremia from other sources (Huang et al. 1996), with periodontal disease being increasingly recognized as a potential source (Crippin and Wang 1992; Ohyama et al. 2009). Most PLA are polymicrobial and mixed enteric and anaerobic species are common pathogens, with one case series identifying anaerobes in up to 45% cases (Nozawa et al. 2011). However, monomicrobial infections are also possible.

*Fusobacterium* is an anaerobic bacterium that is found in the oropharyngeal cavity and is believed to be strongly associated with the formation of dental plaque (Bolstad et al. 1996). PLA secondary to *F. nucleatum* has rarely been reported previously, and mostly in immunodeficient hosts with the exception of a few (Tweedy and White 1987; Scoular et al. 1992; Etienne et al. 2001; Ala et al. 2001; Wells et al. 2005; Schattner and Gotler 2014; Dahya et al. 2015; Ahmed et al. 2015). We report the case of a 69 year-old immunocompetent female who was found to have a pyogenic liver abscess secondary to *Fusobacterium nucleatum.*

## Case description

A 69 year-old female presented with a 2-month history of vague right upper quadrant (RUQ) pain, intermittent fevers and chills. Her pain was dull in nature and was slightly worse with deep inspiration. Her past medical and surgical histories were significant for emphysema, well controlled diabetes mellitus, known pancreatic and renal cysts and an infra-renal abdominal aortic aneurysm (AAA) endovascular repair a few years ago.

**Figure 1:**
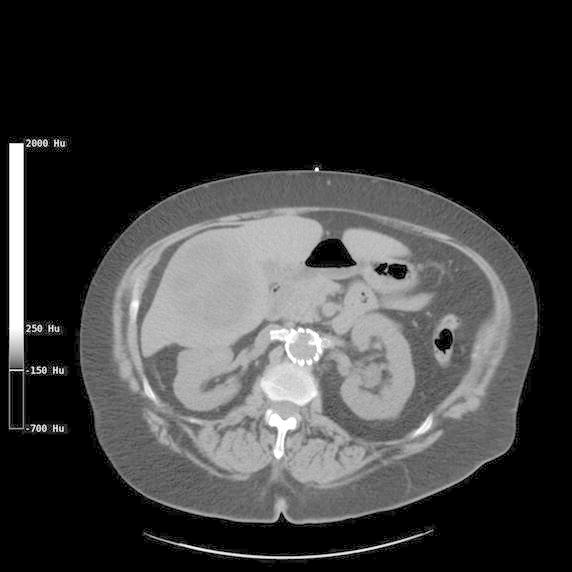
Abdominal computerized tomography (CT) showing a multiseptated thick walled mass in the right hepatic lobe.

**Figure 2:**
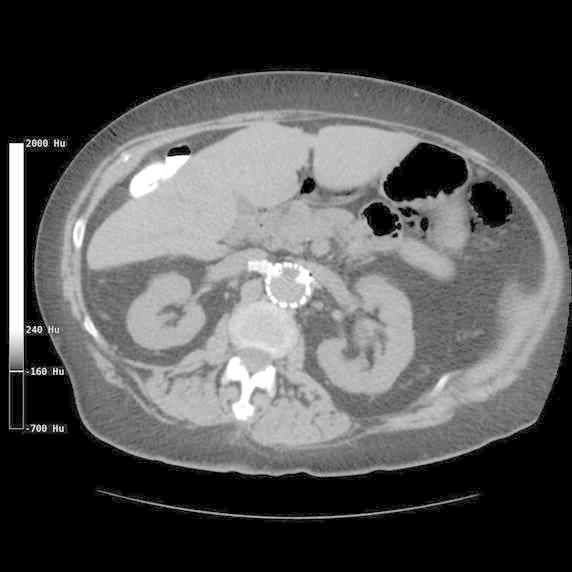
Repeat CT at 4 months post discharge showing complete resolution of the hepatic abscess.

On presentation, her vitals showed a temperature of 38.1°C (100.6°F), heart rate 117 beats per minute, blood pressure 98/62 and an oxygen saturation of 96% on room air with a respiratory rate of 15 breaths per minute. Physical exam revealed poor dental hygiene and tenderness in the abdomen, which was worst in the RUQ. Murphy sign was negative and there were no signs of peritonitis. Labs revealed significant leucocytosis (22.82 × 10^3^/µl) with 94.2% neutrophils. Liver function tests revealed a normal AST and ALT (25 and 22 U/L, respectively) and a normal total bilirubin (1.3 mg/dL). Ultrasound imaging of the right upper quadrant (RUQ), done to investigate the pain, revealed a 9 × 7 × 7 cm hypoechoic density in the right hepatic lobe. Due to concern for possible infection, she was started on broad spectrum antibiotics, Vancomycin 1.5 g daily and Meropenem 500 mg bid. Abdominal computerized tomography (CT) showed a multiseptated thick walled mass in the right hepatic lobe (Figure 1). The CT did not show any other potential sources of infection or malignancy and the previously known pancreatic and renal cysts were stable in size and appearance. Vancomycin was stopped by Day 3 of admission due to low concern for MRSA infection. She underwent CT-guided pigtail drain placement which revealed thick, purulent material. Microscopic examination of the abscess fluid revealed polymorphonuclear leucocytes but gram stain was negative. However, cultures from the drain fluid as well as admission blood cultures (2 out of 2 each) revealed *Fusobacterium nucleatum*. X-rays of the teeth showed evidence of moderate to severe chronic periodontitis for which she underwent extraction of all remaining teeth. She also underwent a colonoscopy to rule out occult colonic malignancy as the incidence of PLA is disproportionately higher in patients with occult colonic malignancy (Qu et al. 2012; Kao et al. 2012; Huang et al. 2012; Lai et al. 2014). The colonoscopy only showed diverticulosis. She improved remarkably and was eventually discharged home on intravenous Ertapenem (chosen over penicillins/cephalosporins due to her allergies) to complete a total of 2 weeks of intravenous antibiotic therapy, followed by a 4 week suppressive regimen of oral Penicillin VK. A repeat CT at 4 months post discharge (Figure 2) showed complete resolution of the hepatic lesion.

## Discussion

Differential diagnoses for a liver hypodensity seen on imaging can be broad and can include an amebic or pyogenic liver abscess, primary or metastatic malignancy (especially colonic). Since the suspicion for PLA was high based on the ultrasound, the patient was started on broad spectrum antibiotic coverage with Vancomycin and Meropenem. The oral cavity was considered as a potential source of infection due to the patient’s poor dental hygiene. However, hematogenous spread of infection leading to PLA, especially from an oropharyngeal source is extremely uncommon and *F. nucleatum* was not initially suspected as the causative organism.

Immunodeficient patients tend to develop opportunistic and superimposed infections, occasionally with rare organisms. Among the sixteen cases of PLA due to *F. nucleatum* that have been reported (including current case), only five patients (31.25%) had documented immunocompetence. Out of these five patients, four (80%) were found to have dental disease and the remaining one patient had evidence of a tonsil infection (Mémain et al. 2001). This probably implies that *F. nucleatum* leading to distant infection and abscess formation is very likely to have originated from the oropharyngeal region and likely secondary to poor dental hygiene leading to dental disease. Other rare organisms that have been known to cause PLA are *Citrobacter koseri* (Gupta et al. 2013)*, Gemella morbillorum* (Borro et al. 2014) and S*treptococcus milleri* (Chen et al. 2012), which is especially known for its underlying association with colorectal neoplasia.

The liver is a major site of intraabdominal abscesses (Altemeier et al. 1973). However, it is not clear why the liver seems to be preferentially affected over other visceral organs by *F. nucleatum*. While this could just be due to the liver’s dual blood supply which is derived from the portal as well as systemic circulations, it is also possible that the formation of liver abscesses in *Fusobacterium* bacteremia represents an early stage of infection and if left untreated, it might lead to involvement of other organs as well. Organism (*F. nucleatum)* and organ (Liver) specific interaction and factors that are still unknown, might have a role to play as well.

Early identification and treatment of PLA is very essential, given the high likelihood of fatality in untreated cases (Stroup et al. 2007). Known risk factors for mortality are presence of anaerobic infection, abscess greater than 5 cm in size and the need for open surgical drainage (Yang et al. 2004; Lok et al. 2008; Chen et al. 2012). Treatment consists of abscess drainage as well as appropriate antibiotic therapy. Like other infections, collection of cultures should ideally be performed prior to initiation of antibiotic therapy.

## Conclusions

We report an unusual case of a PLA due to *F. nucleatum* infection which seems to be an emerging causative organism for PLA. In this case the infection was likely from a dental source and the host was immunocompetent. Poor oropharyngeal hygiene seems to have a major role in infection from this organism and therefore *F. nucleatum* should be considered as a differential of PLA in patients with poor dental hygiene, especially with a known history of immunodeficiency. Radiology guided drainage of the abscess and antibiotic therapy (including anerobic coverage) are the mainstays of therapy and patients rarely require invasive surgery for PLA (Heneghan et al. 2011; Pang et al. 2011).

## Abbreviations

PLA: Pyogenic liver abscess
RUQ: right upper quadrant
CT: computerized tomography
AAA: abdominal aortic aneurysm

## References

[CR1] Ahmed Z, Bansal SK, Dhillon S (2015). Pyogenic liver abscess caused by Fusobacterium in a 21-year-old immunocompetent male. World J Gastroenterol WJG.

[CR2] Ala A, Safar-Aly H, Millar A (2001). Metallic cough and pyogenic liver abscess. Eur J Gastroenterol Hepatol.

[CR3] Altemeier WA, Culbertson WR, Fullen WD, Shook CD (1973). Intra-abdominal abscesses. Am J Surg.

[CR4] Bolstad AI, Jensen HB, Bakken V (1996). Taxonomy, biology, and periodontal aspects of *Fusobacterium nucleatum*. Clin Microbiol Rev.

[CR5] Borro P, Sumberaz A, Testino G (2014). Pyogenic liver abscess caused by Gemella morbillorum. Colomb Médica Cali Colomb.

[CR6] Chen Y-Y, Lee J-C, Yen H-H, Wu S-S, Soon M-S (2012). Pyogenic liver abscess and colorectal neoplasia: a case series. Scand J Infect Dis.

[CR7] Crippin JS, Wang KK (1992). An unrecognized etiology for pyogenic hepatic abscesses in normal hosts: dental disease. Am J Gastroenterol.

[CR8] Dahya V, Patel J, Wheeler M, Ketsela G (2015). *Fusobacterium nucleatum* endocarditis presenting as liver and brain abscesses in an immunocompetent patient. Am J Med Sci.

[CR9] Etienne M, Gueit I, Abboud P, Pons JL, Jacquot S, Caron F (2001). *Fusobacterium nucleatum* hepatic abscess with pylephlebitis associated with idiopathic CD4(+) T lymphocytopenia. Clin Infect Dis Off Publ Infect Dis Soc Am.

[CR10] Gupta M, Sharma A, Singh R, Lehl SS (2013). Citrobacter koseri: an unusual cause of pyogenic liver abscess. BMJ Case Rep.

[CR11] Heneghan HM, Healy NA, Martin ST, Ryan RS, Nolan N, Traynor O (2011). Modern management of pyogenic hepatic abscess: a case series and review of the literature. BMC Res Notes.

[CR12] Huang CJ, Pitt HA, Lipsett PA, Osterman FA, Lillemoe KD, Cameron JL (1996). Pyogenic hepatic abscess. Changing trends over 42 years. Ann Surg.

[CR13] Huang W-K, Chang JW-C, See L-C, Tu H-T, Chen J-S, Liaw C-C (2012). Higher rate of colorectal cancer among patients with pyogenic liver abscess with Klebsiella pneumoniae than those without: an 11-year follow-up study. Colorectal Dis Off J Assoc Coloproctology G B Irel.

[CR14] Kao W-Y, Hwang C-Y, Chang Y-T, Su C-W, Hou M-C, Lin H-C (2012). Cancer risk in patients with pyogenic liver abscess: a nationwide cohort study. Aliment Pharmacol Ther.

[CR15] Kaplan GG, Gregson DB, Laupland KB (2004). Population-based study of the epidemiology of and the risk factors for pyogenic liver abscess. Clin Gastroenterol Hepatol Off Clin Pract J Am Gastroenterol Assoc.

[CR16] Lai H-C, Lin C-C, Cheng K-S, Kao J-T, Chou J-W, Peng C-Y (2014). Increased incidence of gastrointestinal cancers among patients with pyogenic liver abscess: a population-based cohort study. Gastroenterology.

[CR17] Lok K-H, Li K-F, Li K-K, Szeto M-L (2008). Pyogenic liver abscess: clinical profile, microbiological characteristics, and management in a Hong Kong hospital. J Microbiol Immunol Infect Wei Mian Yu Gan Ran Za Zhi.

[CR18] Mémain N, Arvaniti K, Bruneel F, Leport C, Wolff M, Regnier B (2001). Septic shock with liver abscess in an immunocompetence patient. Presentation of an unusual *Fusobacterium nucleatum* infection. Presse Médicale Paris Fr (1983).

[CR19] Mohsen AH, Green ST, Read RC, McKendrick MW (2002). Liver abscess in adults: ten years experience in a UK centre. QJM Mon J Assoc Physicians.

[CR20] Nozawa Y, Joshita S, Fukushima M, Sugiyama Y, Ichikawa Y, Kimura T (2011). A case of pyogenic liver abscess infected with Fusobacterium necrophorum depicted by microscopy and confirmed by tissue culture. Intern Med Tokyo Jpn.

[CR21] Ohyama H, Nakasho K, Yamanegi K, Noiri K, Kuhara K, Kato-Kogoe K (2009). An unusual autopsy case of pyogenic liver abscess caused by periodontal bacteria. Jpn J Infect Dis.

[CR22] Pang TCY, Fung T, Samra J, Hugh TJ, Smith RC (2011). Pyogenic liver abscess: an audit of 10 years’ experience. World J Gastroenterol WJG.

[CR23] Qu K, Liu C, Wang Z-X, Tian F, Wei J-C, Tai M-H (2012). Pyogenic liver abscesses associated with nonmetastatic colorectal cancers: an increasing problem in Eastern Asia. World J Gastroenterol WJG.

[CR24] Rahimian J, Wilson T, Oram V, Holzman RS (2004). Pyogenic liver abscess: recent trends in etiology and mortality. Clin Infect Dis Off Publ Infect Dis Soc Am.

[CR25] Schattner A, Gotler J (2014). Fever, night sweats, and abnormal liver enzymes. Lancet.

[CR26] Scoular A, Corcoran GD, Malin A, Evans BA, Davies A, Miller RF (1992). Fusobacterium nucleatum bacteraemia with multiple liver abscesses in an HIV-I antibody positive man with IgG2 deficiency. J Infect.

[CR27] Stroup JS, Depriest KL, Haraway GD (2007). Fusobacterium pyogenic liver abscess. Infect Med.

[CR28] Tweedy CR, White WB (1987). Multiple Fusobacterium nucleatum liver abscesses. Association with a persistent abnormality in humoral immune function. J Clin Gastroenterol.

[CR29] Wells CD, Balan V, Smilack JD (2005). Pyogenic liver abscess after colonoscopy in a patient with ulcerative colitis. Clin Gastroenterol Hepatol Off Clin Pract J Am Gastroenterol Assoc.

[CR30] Wong W-M, Wong BCY, Hui CK, Ng M, Lai KC, Tso WK (2002). Pyogenic liver abscess: retrospective analysis of 80 cases over a 10-year period. J Gastroenterol Hepatol.

[CR31] Yang C-C, Yen C-H, Ho M-W, Wang J-H (2004). Comparison of pyogenic liver abscess caused by non-Klebsiella pneumoniae and Klebsiella pneumoniae. J Microbiol Immunol Infect Wei Mian Yu Gan Ran Za Zhi.

